# Lymphoepithelial Carcinoma of the Nasal Cavity With EBV Infection in a North African Man

**DOI:** 10.4021/wjon2010.04.204w

**Published:** 2010-04-30

**Authors:** Amel Trabelsi, Sameh Tebra, Soumaya Ben Abdelkrim, Nadia Beizig, Ahlem Bdioui, Faten Hammedi, Noureddine Bouaouina, Moncef Mokni

**Affiliations:** a Department of Pathology, Farhat Hached hospital, 4000, Sousse, Tunisia; b Department of Radiotherapy, Farhat Hached hospital, 4000, Sousse, Tunisia

**Keywords:** Lymphoepithelial carcinoma, EBV, Nasal cavity, Pathology

## Abstract

We report the case of a 58-year-old Tunisian man who presented with a 2 months' history of left nasal obstruction and one episode of epistaxis. Nasal endoscopy revealed a polypoid mass of the left nasal septum. Magnetic resonance imaging showed a left nasal cavity tumor with erosion of the orbit. Diagnosis of nasal cavity lymphoepithelial carcinoma EBV positive was performed on biopsy. The patient was treated by chemotherapy and radiotherapy. No tumor recurrence has been reported with a follow-up of 12 months.

## Introduction

Lymphoepithelial carcinoma of the nasal cavity (LEC NC) is an extremely rare tumor, with most cases identified in patients from Southeast Asian countries [1]. Similar to its morphological analogue of the nasopharynx, LEC NC has a strong association with EBV [2]. We report a new case and discuss the diagnostic problems of this rare entity.

## Case Report

**Figure 1:**
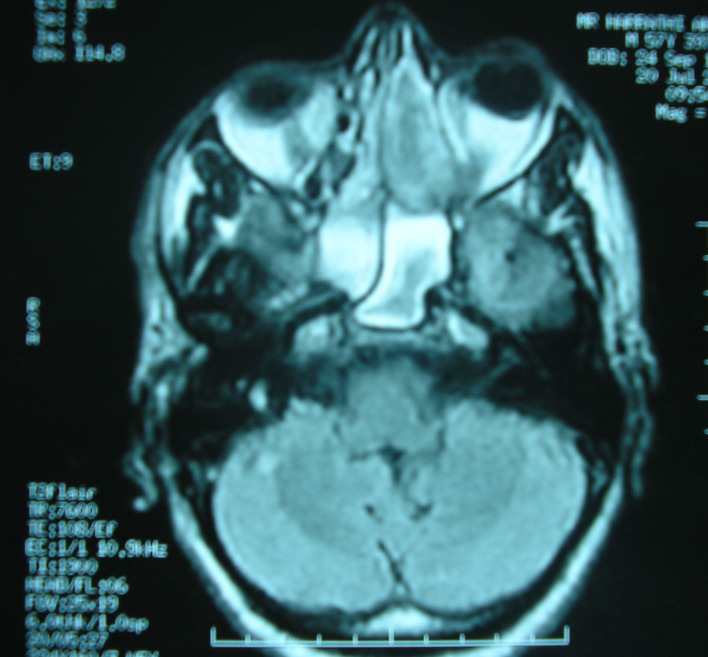
MRI of nose and paranasal sinuses: nasal cavity tumor with erosion of the left orbit.

**Figure 2:**
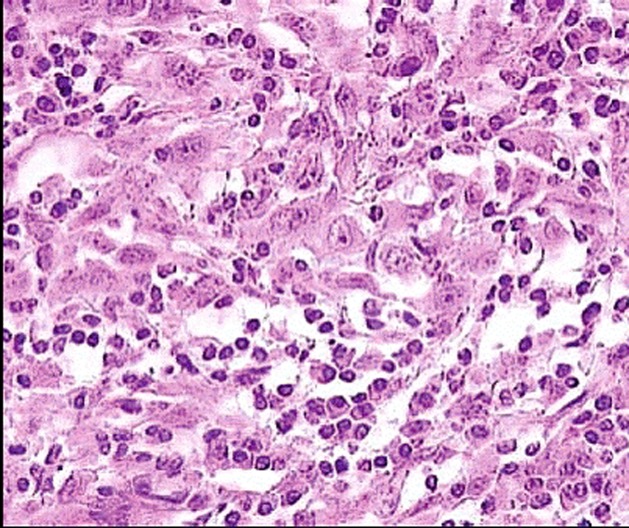
Malignant cells showing a syncitial appearance (HE x 200).

**Figure 3:**
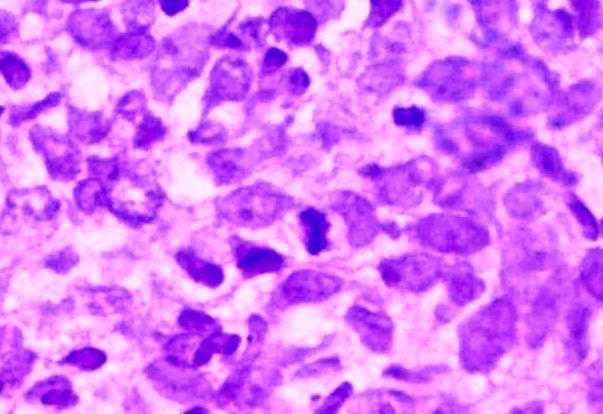
Anaplastic cells with vesicular nuclei and prominent nucleoli (HE x 400).

**Figure 4:**
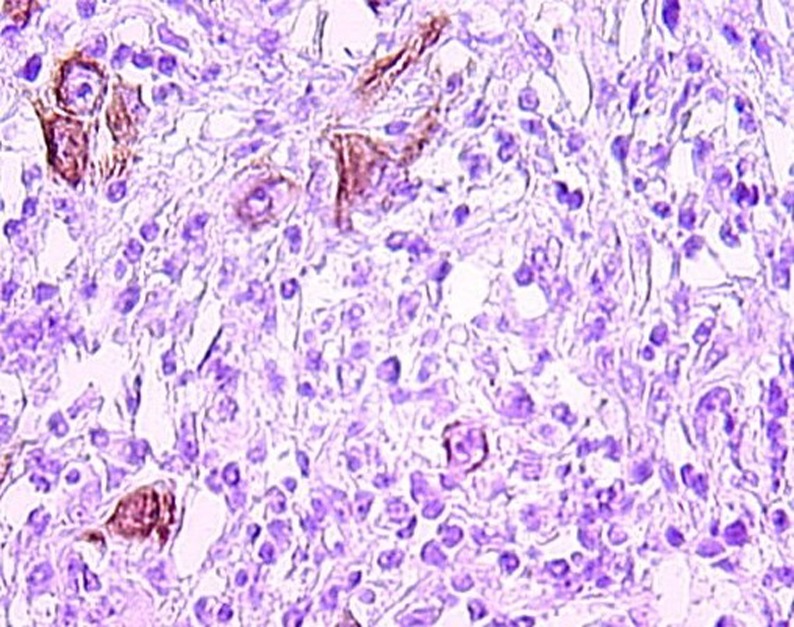
Tumor cells immunoreactivity with EMA antibody (IHC x 400).

A 58-year-old Tunisian man with no medical history presented with a two months' history of left nasal obstruction and one episode of epistaxis. Nasal endoscopy revealed a smooth reddish polypoid mass of the nasal septum. The mass bled easily whenever palpated by the instrument. There was no evidence of lymph node metastasis. Magnetic resonance imaging (MRI) showed a 30 x 50 mm left nasal cavity tumor with erosion of the orbit (Fig. 1). The tumor was found to originate from the nasal septum. Histological examination of the biopsy demonstrated a tumor proliferation that was made of irregular sheets, islands and single neoplastic cells richly infiltrated by lymphocytes and plasma cells. Epithelial component consisted of large cells with indistinct cell borders resulting in syncitial appearance (Fig. 2), vesicular nuclei and moderate mitotic activity (Fig. 3). Necrosis and keratinization were absent. Immunohistochemically, most of tumor cells were positive for cytokeratin and epithelial membrane antigen (EMA) (Fig. 4). The surrounding cellular infiltrate was a mixture of CD20 and CD3 positive B and T lymphocytes. Immunohistochemical expression of EBV latent membrane protein 1 (LMP1) and in situ hybridization to EBV encoded RNA were positive. The diagnosis of primary LEC NC EBV positive was performed after excluding metastatic nasopharyngeal undifferentiated carcinoma of the nasal cavity by random biopsies from the nasopharyngeal mucosa. The tumor was classified T4N0M0. Systemic chemotherapy was started and consisted in intravenous Adriamycin and Cisplatin. Radiotherapy was done based on 72 Gy in the tumor and 52.2 Gy in the bilateral cervical lymph node region: 1.8 Gy/day, 5 days per week. The patient remains alive and disease free 12 months after treatment.

## Discussion

Lymphoepithelial carcinoma of the nasal cavity is extremely rare with the majority of the cases identified in Southeast Asian countries [1]. It was recently accepted as a distinct entity separated from nasopharyngeal carcinoma by topography but still similar to this entity histologically [3, 4]. In the past, LEC and undifferentiated carcinoma of the sinonasal tract have been used as synonyms, but the latter is more aggressive [2]. Clinically, most patients presented with nasal obstruction and/or epistaxis. Occasionally, cervical lymph nodes' metastases may be the initial presenting symptoms [1, 3]. The nasal cavity is affected most often, followed by the paranasal sinuses [1]. Morphologically, LEC NC is similar to nasopharyngeal undifferentiated carcinoma. The tumor is arranged in irregular islands, solid sheets and single neoplastic cells with syncitial appearance. The nuclei are usually vesicular with prominent nucleoli. The lymphoid component tends to be less prominent as compared with its nasopharyngeal counterpart. Mitoses may be seen but necrosis and keratinization are usually absent [2]. Immunohistochemically, tumor cells show an immunoreactivity for pancytokeratin and EMA. The surrounding cellular infiltration is a mixture of CD20 and CD3 positive B and T lymphocytes. LEC NC must be distinguished from other malignant tumors particularly sinonasal undifferentiated carcinoma, lymphoma and malignant melanoma seeing that each lesion has a different treatment and outcome [3]. Immunohistochemical stains allow distinction from lymphoma and melanoma. Sinonasal undifferentiated carcinoma is EBV-negative and tends to be much more pleomorphic with central necrosis and high mitotic index [2]. Similar to its nasopharyngeal counterpart, LEC NC has a strong association with EBV, detected by immunohistochemistry and in situ hybridization technique for EBV-encoded RNA (EBER). Because of the reduced number of the reported cases, it is difficult to determine the optimal treatment of LEC NC. This tumor is highly radiosensitive and radiotherapy should be considered as the main treatment even when there is lymph node metastasis [5]. Chemotherapy may be added particularly when there are distant metastases [2]. More recently, intensity modulated radiation therapy seems to provide low rates of radiation-induced toxicity with high local control and better survival [6, 7]. The prognosis of LEC NC is stage-dependent, it declines when distant metastases are present [2].
